# Fabrication and characterization of AlN-based flexible piezoelectric pressure sensor integrated into an implantable artificial pancreas

**DOI:** 10.1038/s41598-019-53713-1

**Published:** 2019-11-20

**Authors:** M. A. Signore, G. Rescio, C. De Pascali, V. Iacovacci, P. Dario, A. Leone, F. Quaranta, A. Taurino, P. Siciliano, L. Francioso

**Affiliations:** 1 CNR-IMM Institute for Microelectronics and Microsystems, Via Monteroni, 73100 Lecce, Italy; 2 BioRobotics Institute and Department of Excellence in Robotics & AI, Scuola Superiore Sant’Anna, 56025 Pontedera, Italy

**Keywords:** Sensors and biosensors, Metals and alloys

## Abstract

This study reports on the fabrication and characterization of an event detection subsystem composed of a flexible piezoelectric pressure sensor and the electronic interface to be integrated into an implantable artificial pancreas (IAP) for diabetic patients. The developed sensor is made of an AlN layer, sandwiched between two Ti electrodes, sputtered on Kapton substrate, with a preferential orientation along c-axis which guarantees the best piezoelectric response. The IAP is made of an intestinal wall-interfaced refilling module, able to dock an ingestible insulin capsule. A linearly actuated needle punches the duodenum tissue and then the PDMS capsule to transfer the insulin to an implanted reservoir. The device is located at the connection of the needle with the linear actuator to reliably detect the occurred punching of the insulin-filled capsule. Finite Element Analysis (FEA) simulations were performed to evaluate the piezoelectric charge generated for increasing loads in the range of interest, applied on both the sensor full-area and footprint area of the Hamilton needle used for the capsule punching. The sensor-interface circuit was simulated to estimate the output voltage that can be obtained in real operating conditions. The characterization results confirmed a high device sensitivity during the punching, in the low forces (0–4 N) and low actuator speed (2–3 mm/s) ranges of interest, meeting the requirement of the research objective. The choice of a piezoelectric pressure sensor is particularly strategic in the medical field due to the request of self-powered implantable devices which do not need any external power source to output a signal and harvest energy from natural sources around the patient.

## Introduction

The challenge to treat diseased or failing human organs has represented the driving force behind significant research efforts. Fully artificial medical devices consisting in non-biological materials, i.e. artificial organs^1^, and aimed at replacing or augmenting lost natural organs functionalities, gained particular attention in this framework. The technology involved in the artificial organs realization is very extensive and multidisciplinary. The combination of several scientific and technological areas – i.e. biological sciences, micro/nanotechnology, cognitive sciences, information technology, materials science and sensor technology - can be considered the real protagonist of such a revolution in the medical field. In particular, sensor technology is one of the most relevant areas because the integration of sensors into an artificial organ permits the control of the implanted organ functions, which represents one of the major practical problems to solve, aimed to the monitoring of the device working to guarantee its reliability. The use of microfabricated flexible implantable sensors represents, today, a promising and innovative approach to potentially manufacture artificial organs^2^.

Among the multitude of flexible devices, wearable and lightweight pressure sensors play an important role because they are fundamental electronic components in many fields of application^3,4^. In particular, in the biomedical field, they could be employed in implantable devices, i.e.for monitoring the intracranial pressure during neurosurgery, the pressure in respiratory diseases treatment, the blood pressure during surgical procedures, the intrauterine and abdominal/urinary pressure in obstetrics and urology, respectively^5^.

Among the different classes of pressure sensors (i.e. piezoelectric, capacitive, resistive), the piezoelectric ones represent an interesting type of active sensors whose operation does not rely on any form of external powering. The development of self-powered devices able to harvest energy from the surrounding and from other fisiological processes is one of more ambitious goal in the field of implantable artificial organs since powering is one of the main bottlenecks in implantable devices. In this sense such kind of sensors could represent the response^6^.

Piezoelectric materialsallow to convert mechanical energy into electrical signals (and vice versa), thus represent an attractive building block forautonomous pressure sensors y exploiting the correlation between strain and voltage/current. Beside the self-powering characteristic, piezoelectric pressure sensors exhibit many other advantages which are required for a reliable monitoring of the physiological parameters in the human body. As an example, they enable to monitor a wide range of pressure with suitable resolution allowing to discriminate between clinically normal and abnormal conditions and to derive information on the medical treatment^7^; they can be fabricated in very small size and simple geometries for an easier implantation into human body^8,9^; they can be easily realized on flexible substrate, then they can be bent, stretched, or twisted to adapt to the local tissue geometry improving conformal contact with the physiological environment^10^.

In spite of smaller piezoelectric constants in comparison to the well-known PZT and ZnO piezoelectric materials employed for sensor applications, aluminum nitride (AlN) appears extremely interesting for its peculiar low dielectric constant (ε ≈ 9–11), low dielectric losses, high electromechanical coupling coefficient, high temperature/humidity stability, high signal-to-noise ratio. Furthermore, it can be processed and deposited at low temperatures, by well-consolidated techniques (i.e. sputtering), preserving the compatibility with standard microfabrication technologies. The possibility to integrate AlN thin films on flexible polymer substrates, allows to provide microsystems with nnovative sensing and actuation capabilities^11,12^ and and enables to overcome some of the limitation of conventional stiff substrates^13^. Moreover, AlN is a non-toxic and biocompatible material - which results suitable for devices implanted in the human body for medical use, as it will be reported in this paper. Therefore, the easiness of both piezoelectric AlN thin film production and integration on different kind of substrates, permits to overcome the typical issues preventing clinical applications, such as i) the dependence on complex cleanroom fabrication tools and high temperature processes, ii) the use of battery to power the device and iii) the use of toxic precursors in the production process^14^.

In this paper the authors describe the fabrication and characterization of a complete system composed of a flexible AlN-based piezoelectric pressure sensor operating in d_33_-mode for quasi-static measurements, and a charge mode amplifier interface. It has been designed to be integrated into a needle movement stage to assist in a reliable way specific operational phases when included into an implantable artificial pancreas (AP). In particular, the target AP^15^ system is based on an innovative refilling strategy based on injestible pills. The devised refilling module is interfaced with the intestine walland includes a capsule docking mechanisms and a linearly actuated needle aimed at punching the capsule and at transferring the insulin to the internal reservoir. The AP, so as it has been designed, requires a sensor to monitor needle insertion into the capsule. Many of the various solutions that could be considered, as fiber optic or capacitive pressure sensor, result disadvantageous for this specific application. As an example, the fiber optic approach is costly, hard to integrate and requires light sources with huge power consumption; capacitive approach may be criticized from the point of view of parasitic capacitances introduced when the needle is in contact with duodenum tissue with high water content or gastric secretions. With respect to other similar solutions, a piezoelectric pressure sensor presents numerous advantages, such as the simple architecture, fabrication easiness, self-powering which is an important feature for implantable devices, and energy saving. The designed piezoelectric pressure sensor, is devised to be placed at the interface between the linear actuator and the needle. The sensor should detect the correct punching of the capsule and provide a feedback signal to the main control electronic unit. A simple sensor signal analysis (output waveform) can identify the events related to the punching of the ingestible capsule. The fabricated device is sufficiently flexible and sensitive to low forces (0–4 N) and low actuator speed (2–3 mm/s), which represent the ranges of interest, fulfilling the requirement of our research objective. The low actuation speed represents a design specification motivated to limit the capsule wall damage and avoid capsule release from magnetic holder.

Room temperature deposition of piezoelectric AlN thin film on a Kapton substrate with (0002) orientation was successful obtained in this work, achieving a result that is very difficult to obtain without additional energy. Removing the substrate heating from the deposition process, while keeping the quality of the film generally obtained with high temperature processes, is a key challenge for the improvement of the devices where thin films are integrated. Furthermore, the deposition at room temperature is completely compatible with photolithography, because it avoids problems of photoresist degradation and film contamination related to high temperature process.

## Results and Discussion

### Morphological and structural characterization of the AlN active layer

**Figure 1:**
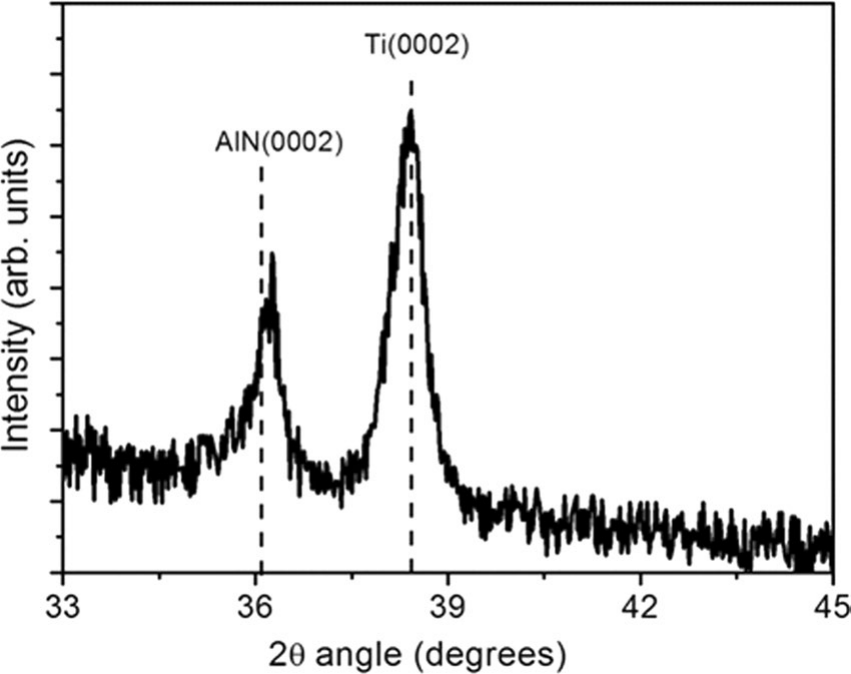
XRD spectrum of the bilayer Ti(150 nm)/AlN(500 nm) sputtered on Kapton substrate.

A thin film of AlN has been employed as active material in the designed piezoelectric device. Piezoelectricity is an anisotropic effect, closely related to material symmetry at the microscopic level. A non-centrosymmetric lattice with charge separation and unbalanced distribution is needed to generate piezoelectric response. Among the 32 possible classes of symmetry, piezoelectric crystals are found in 20 non-centrosymmetrical ones (i.e. wurtzite). Aluminum nitride has a wurtzite structure included in the hexagonal 6mm-class of symmetry. In this structure, each Al (N) atom is surrounded by four N (Al) atoms thus creating a tetrahedral structure^16^. Three short-bonded N (Al) atoms (B1 bond) are placed on the c-plane while a long-bonded atom (B2 bond) is located along the c-axis (in the vertical direction). The c-axis is the polar-axis and the wurtzite crystal structure is non-centrosymmetric only along the c direction. Thus, AlN exhibits its highest piezoelectric response, when its growth axis coincides with the (0002) crystalline direction. The bond energy of B2 (vertical direction along the c-axis) is smaller than that of B1 and the surface energy of the (0002) face is a little lower than that of the other faces. Suitable sputtering growth conditions facilitate the arrangement of the target atoms, arriving on the substrate, in the wurtzite structure, with preferential orientation along c-axis, orthogonal to the substrate surface, with the lowest surface energy. XRD analysis is aimed to verify the preferential orientation of the nitride film along the c-direction. Figure 1 shows the X-ray diffraction spectrum of the bilayer AlN/Ti bottom electrode sputtered on Kapton substrate The presence in the spectrum of the (0002) peak confirms the preferential orientation of the nitride film. A slight shift of the peak towards higher 2θ angle respect to the bulk 2θ = 36.03° value can be noted, indicating a film growth under tensile stress (in-plain compressive strain) due to unit cell distortion. By using the Bragg’s law (nλ = 2d sin θ), the lattice constant (c) can be directly calculated and it is equal to 4.935 Å, much lower compared to the bulk value (4.979 Å). As a result, the ratio c/a ∼1.6 for wurtzite phase is no longer maintained and the crystal lattice results elongated along a-axis. Consequently, the lattice parameter in the basal plane is stretched to keep constant the unit cell volume and this cell distortion results in a tensile stress. The biaxial stress can be calculated taking into account the c-axis strain (evaluated from XRD (0002) peak position) and the theoretical stiffness tensor of AlN. It is about 1.5 GPa, similar to values already reported in literature for sputtered AlN thin films^17^. Obviously, this is an approximation based on the hypothesis that the peak position shift is only due to film’s strain. It is known that there are other factors which influence the shift^18^ that have not been considered in this preliminary study.

**Figure 2:**
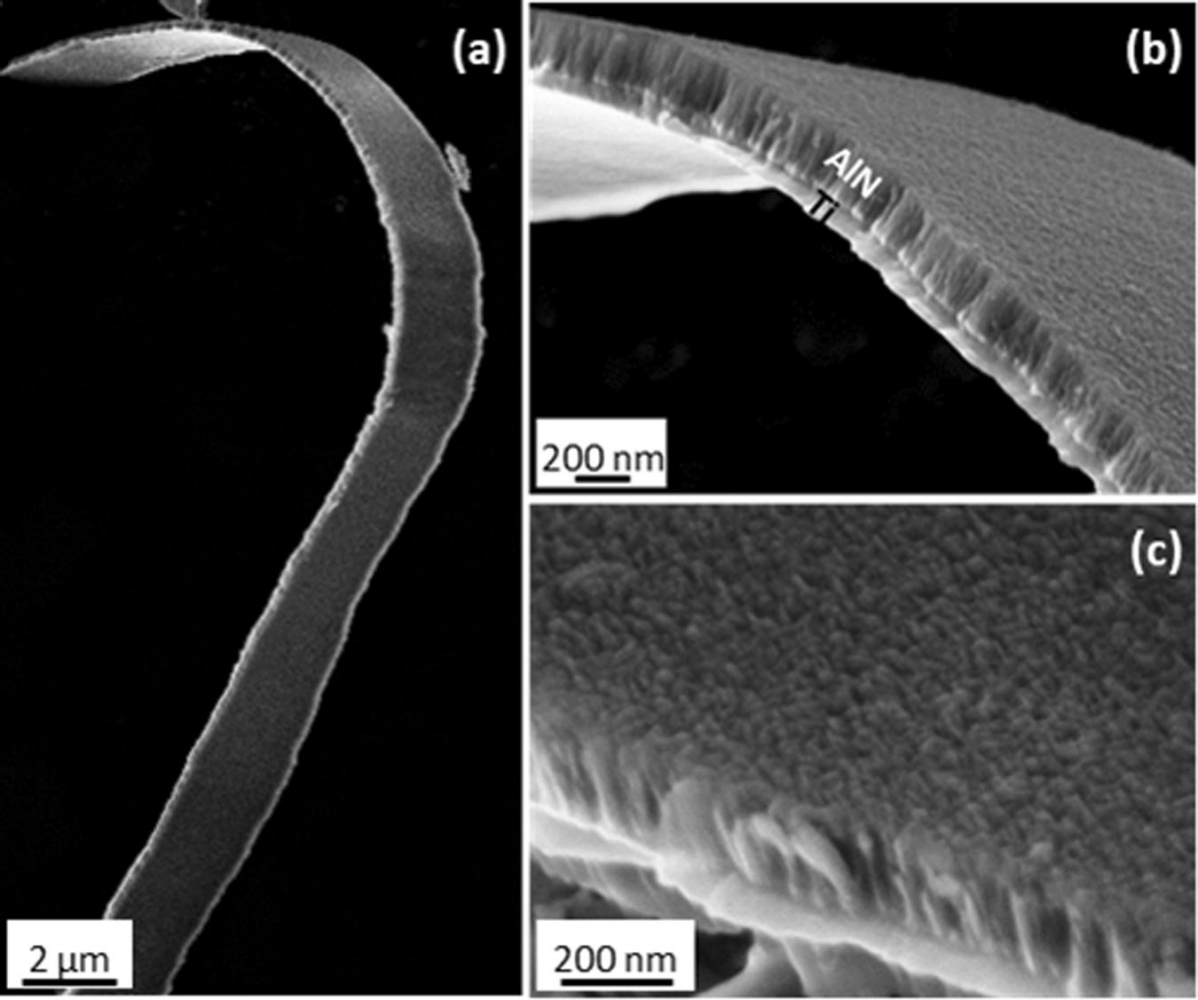
SEM images of a representative Ti/AlN bilayer sputtered on Kapton substrate (AlN film is 250 nm-thick), evidencing (**a**) the high flexibility of the film as well as (**b**,**c**) the cross-sectional and surface morphology.

Figure 2 shows a sequence of SEM images of the Ti/AlN bilayer observed from different orientation and magnification. In Fig. 2a, a long and strongly bent strip, resulting from the preparation procedure described above, is shown. It is worth noting that the bilayer is capable to sustain bending without cracking phenomena, as also evident from the higher magnification image in Fig. 2b, This well remarks the flexibility of the film, essential requirement for its integration into the final piezoelectric device. The cross-sectional and surface morphology can be observed in Fig. 2b,c, where the columnar and dense structure of the film can be observed in detail.

### FEA Simulation results

**Figure 3:**
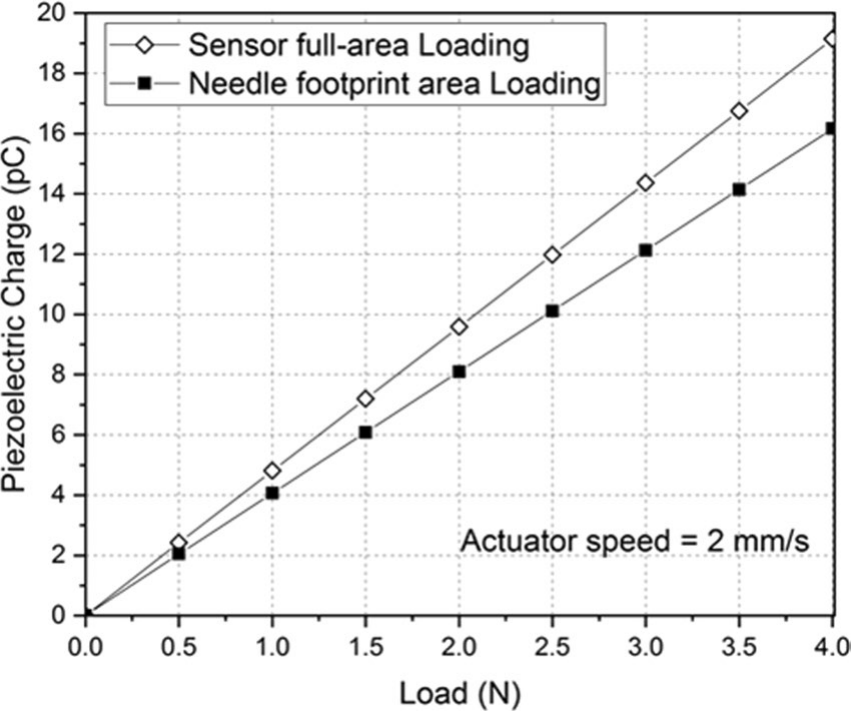
Piezoelectric charge versus increasing normal load, for sensor full-area and needle footprint area loading, evaluated at actuator speed equal to 2 mm/s.

FEA simulations were used to calculate the generated piezoelectric charge while increasing the applied normal force, as a function of the two different loading area: the full-area of the piezoelectric sensor and the footprint area of the Hamilton needle chosen to punch the insulin capsule in the final prototype (400 μm diameter) (Fig. 3). The piezoelectric charge variation of the AlN film thickness was lower than 3% for values in the range 0.5–1.5 μm, which is the most suitable range for better film quality. Therefore, the thickness has been fixed at the lowest value (0.5 μm) which guarantees a better control of the structure quality, decreasing the probability of cracks formation^19^.

**Figure 4:**
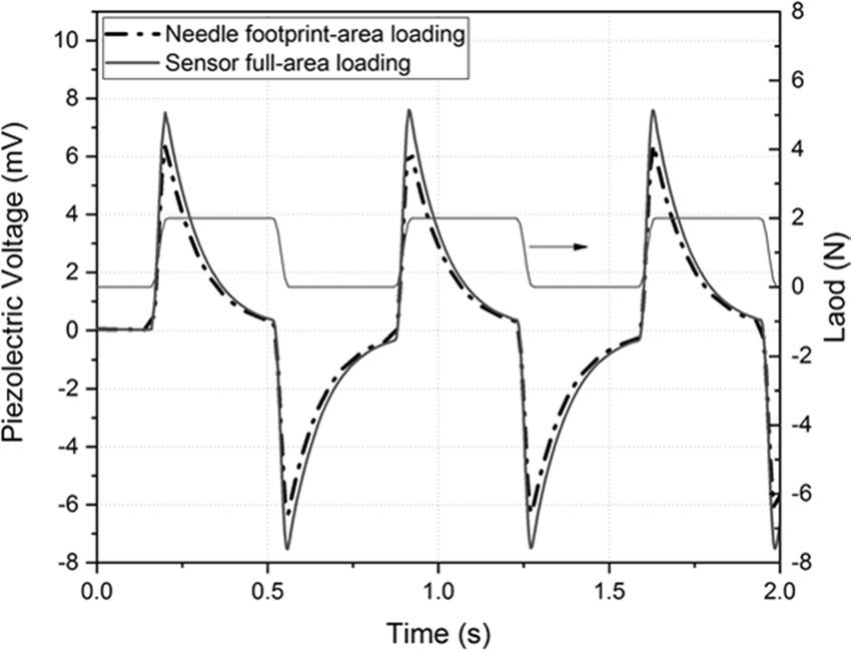
Time evolution of the voltage generated by the AlN based pressure sensor in response to a wave load applied on the sensor full area and on the needle footprint area.

The piezoelectric response of the AlN based sensor to a time-varying normal load was also simulated. For this analysis, the electro-mechanical model was coupled to an interface circuit, in order to assess by FEA the output voltage waveforms of the piezoelectric sensor and charge conversion/amplification system. Figure 4 shows the piezoelectric voltage (measured at the output of the interface circuit) in response to a square wave alternating load of amplitude 2 N, duty cycle 50% and actuator speed of 2 mm/s. The piezoelectric voltage shows a positive pulse within the rise time of the load signal, then it decreases to zero until the load is retained. A negative voltage pulse is again generated when the load signal turns off. A similar trend was also observed experimentally, and an explanation will be given later.

### Fabrication and characterization of the piezoelectric pressure sensor by quasi-static method

**Figure 5:**
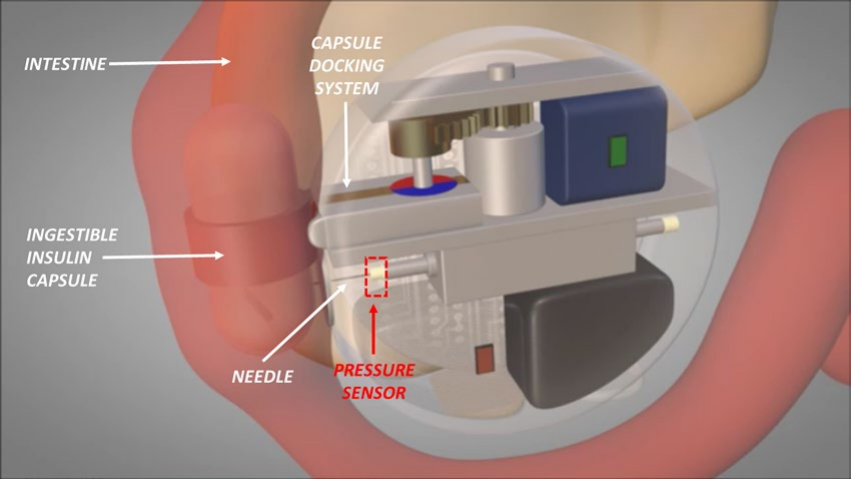
Implantable artificial pancreas prototype; the piezoelectric pressure sensor is located at the connection of the needle with a linear actuator to sense the occurred capsule punching (red rectangular area).

The working principle of a piezoelectric pressure sensor is based on the temporary electric charge generation when it is deformed by an applied external mechanical stress. As previously reported, in this work the piezoelectric pressure sensor was designed to be integrated into an implantable AP whose prototype is depicted in Fig. 5^20^; the red rectangle defines the area where the pressure sensor should be located.

**Figure 6:**
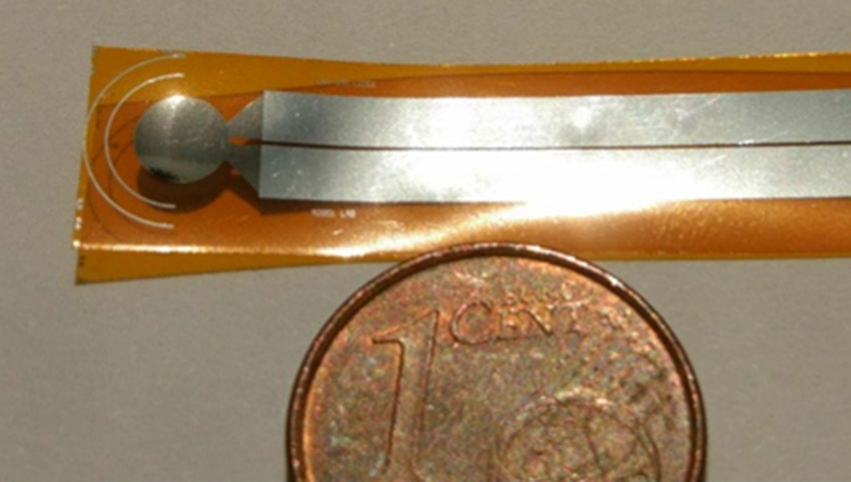
Image of the realized pressure sensor on Kapton substrate.

The prototype comprises a refilling module, interfaced with intestine wall, able to dock an ingestible insulin capsule (Fig. 5, left side). A linearly actuated needle, moving with a speed in the range of 2–3 mm/s, punches the capsule to transfer insulin to an implanted reservoir. At such low speed guarantees a better control of the mechanical actuators involved in the punching-refilling action. The actuator frequency was set in accordance with the specific design requirements; in fact, the low actuator speed has been chosen to limit the capsule wall damage, hole broadening during punching and avoid capsule release event from magnetic holder. The designed piezoelectric pressure sensor is inserted at the connection of the needle with the linear actuator to provide a feedback of the occurred capsule punching mechanism, by measuring a dynamic force variation. Figure 6 shows the fabricated device and the bending property of the sensor is well emphasized. Two piezoelectric modes, namely d_31_ or d_33_, are commonly used in piezoelectric microdevices which can be distinguished according to the relative directions of the electric field and the strain: d_31_ when the electric field is perpendicular to the input strain, and d_33_ when they are parallel^21^. In this case, the sensor operates in d_33_ mode.

The effective active circle area of the pressure sensor (equal to 11.34 mm^2^) is placed right under the rubber rod, used to apply a controlled force to the device. The output signals are then filtered and elaborated by MATHWORKS MATLAB software^22^.

**Figure 7:**
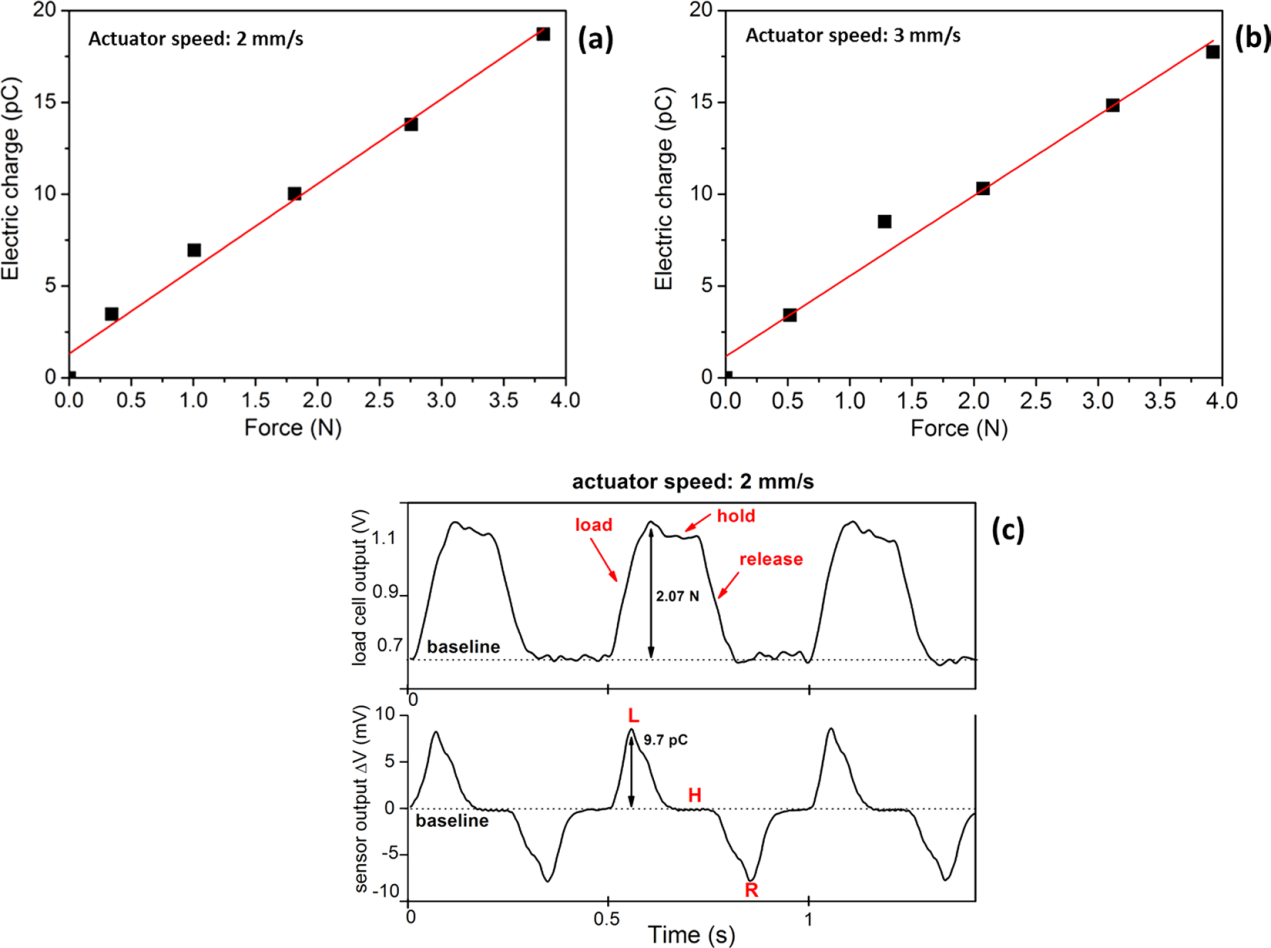
Electrical output vs. force applied on the active area of the piezoelectric sensor when varying actuator speedbetween, (**a**) 2 mm/s and (**b**) 3 mm/s; (**c**) a representative dynamic response at fixed applied force (2.07 N) at 2 mm/s actuator speed.

The sensing performance of the piezoelectric pressure sensor was demonstrated by applying periodic vertical force, ranging from 0 N up to 4 N, to the top of the device in the vertical direction. The relationship between output electric charge and applied force, at two different actuator speeds (2 mm/s and 3 mm/s), is depicted in Fig. 7a,b, respectively. The force values range is obtained by tuning the stroke of the probe (60–240 μm) at a constant speed.

Due to the fact that applied force is related to the stroke of the rubber tip, it’s better to discuss in terms of probe speed instead of frequency (between impacts); in fact the speed of the tip can be tuned within a suitable range (0.1–5 mm/s) while the resultant frequency was constrained in a small range (due to longer stroke required for larger force).

The generated electric charge is consistent with the load cell signal, and it increases proportionally with increasing load. The fitting line (Fig. 7) shows clearly that the piezoelectric sensor has a satisfactory linearity in the range of interest within the limits of experimental error (6%) and the square of correlation coefficient R2 > 0.96. Figure 7c depicts the dynamic response of the sensor by applying a force equal to about 2 N at actuator speed of 2 mm/s. Load cell and sensor output behavior can be correlated^23^. When a vertical compressive stress is applied on the device (“load” in Fig. 7c, upper graph), the AlN thin film undergoes a compressive strain inducing an electric polarization. As a consequence, positive and negative charges accumulation occurs on the two opposite electrodes (top and bottom) and a piezoelectric potential is generated, as observed by the recorded sharp positive voltage peak (“L” in the figure). At constant load (“hold”), no response from the sensor is observed (indicated with “H” in the figure). In this phase, the load is constant and no response is recorded from the sensor; free carriers featured by opposite polarity are attracted from the external circuit to the thin film surfaces, at the electrodes in orderto balance the piezo-potential. Therefore, the voltage output of the device decays to zero, when the potential is equilibrated. During the strain release (“release”), the charges generated by the piezoelectric thin film return in the opposite direction, resulting to a reverse polarity, thus an output negative voltage signal is recorded, which rises up to zero when the free carriers return to the equilibrium state. Therefore, continuously applying and releasing the compressive stress results the alternating voltage.

The d_33_ piezoelectric constant value of the active thin film can be calculated from the average slope of the fitting straight line of Fig. 7 and it results equal to (4.5 ± 0.3) pC/N, in accordance with the range values reported in literature for AlN thin films deposited on rigid substrate, i.e. silicon (4.5–5.3 pC/N)^24^. But, in our case, it should be noted that the piezoelectric coefficient has been experimentally obtained from thin films deposited on flexible substrate which is generally lower than those measured on rigid one (i.e. 2.6 pC/N for AlN thin films on polymer substrate respect to about 5 pC/N on silicon). Therefore, d_33_ value obtained for our AlN thin film grown on Kapton substrate is higher than some values reported in literature^25,26^. Generally, piezoelectric coefficient values, obtained from films grown on flexible substrates, are very often lower than those measured on rigid ones and this is commonly ascribed to a lower active film quality on polymer substrate which makes worse the piezoelectric properties^27^. It is interesting to observe that the fabricated piezoelectric pressure sensor exhibits a good performance even at such a low actuator speed selected for the present study (2 mm/s).

**Figure 8:**
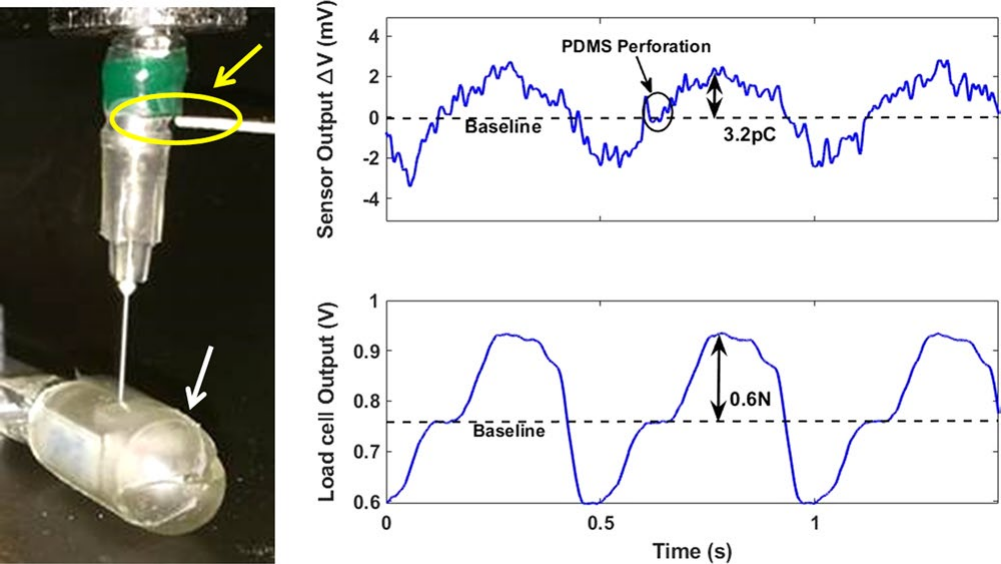
Functional characterization of the piezoelectric pressure sensor in working conditions; the yellow arrow indicates the assembly insertion of the fabricated device while the white arrow shows where the PDMS capsule mounted on the load cell.

The output signal generation of the fabricated device was evaluated also in real working conditions, typical of this specific application. In particular, different sensors have been mounted at the connection of the needle with the shaft of the linear actuator, as indicated in Fig. 8 by the yellow arrow. In this configuration, the needle (gauge 27, 0.41 mm diameter size) punches the PDMS ingestible capsule (indicated by the white arrow in the figure) which will be filled with insulin.

In this experiment, the needle tip displacement was set at constant value of 4 mm and it is justified by the PDMS capsule sizes, 1 mm-thick wall with a diameter of 12 mm; in this way, the needle punches completely the capsule and then it moves forward for 3 mm for the insulin refilling mechanism. The feed speed was increased from 2 mm/s to 3.5 mm/s to find the optimal speed value which supplies the highest electric charge amount. Although the speed variation, the evaluated PDMS punching force was always the same, equal to 0.6 N, and, consequently, the measured output electric charge amount was the same, equal to 3.2 pC (in line with the calculated piezoelectric coefficient). This result does not impose any constraints about the choice of the linear actuator feed speed; this aspect will allow for the best trade-off between insulin transfer procedure speed and optimization of whole AP power consumption. The Signal-to-Noise Ratio (SNR) of the sensor for this specific application was measured as the ratio between the average of the maximum voltage changes due to the needle punches and the standard deviation of the baseline as reported in^28^. The calculated SNR, analyzing the aforementioned experiments of the IAP, was about 6.21. This value is comparable with similar flexible pressure sensor presented in literature^29^ and it is high enough to detect the PDMS capsule perforation. A comparison between the experimental results in Fig. 7 (rubber tip actuation) and Fig. 8 (real capsule punching) highlights the different output waveforms of the sensor: in the first case, the fast separation between the tip and sensor during the ascent phase leads to a sharp signal recovery after the “R” point (Fig. 7); in the capsule case instead, the needle keeps the contact with the capsule wall during the ascent phase for about 1.5 s, leading to a smoothed recovery signal. These different output waveforms will be usefully exploited in the future for the recognition of different materials punching (duodenum, capsule wall) during the needle stroke.

The figure of merit (FOM) typically adopted for piezoelectric devices is given by the formula FOM = e_31_2/ɛ_0_ɛ_r_^30^. Considering a value of experimental piezoelectric constant e_31_ = −0.7 ± 0.1 C/m^2^, experimental ɛ_r_ = 10.55 ± 0.25, experimental Young modulus E = 293 ± 15 GPa, the calculated FOM for our device is 4.7 ± 1 GPa. Such value reports lower than FOMs (from 9 to 12 GPA) on published papers for AlN-based devices^31,32^, but these values are generally not comparable to present work due to a different growth temperature, doping presence or silicon substrate adoption.

## Methods

### Deposition and characterization of thin films

Ti/AlN/Ti trilayer has been sputtered on a Kapton substrate from high-purity (99.999%) Ti and Al targets, 4″ diameter. The target-substrate distance was fixed at 80 mm. The base pressure in the vacuum chamber, before the deposition process, was 2 × 10^−7^ mbar. Ti layers have been deposited in Ar atmosphere at a pressure of 2.5 × 10^−2^ mbar while AlN in (Ar + N_2_) mixture at a pressure of 4.3 × 10^−3^ mbar with N_2_ flux percentage $\frac{F({N}_{2})}{F({N}_{2})+F(Ar)}$ equal to 60%. The RF power applied to both targets for plasma ignition was 150 W. All depositions have been performed at room temperature, particularly challenging for the compatibility of the growth process with all involved microfabrication steps for the device realization. Ti and AlN films thicknesses were fixed at 150 nm and 500 nm, respectively, and Kapton substrate was 50 μm-thick.

X-ray diffraction measurements (XRD) based on Cu-Kα radiation in the θ–2θ configuration, enabled to study the structural properties of the films.

On the other hand, scanning electron microscopy (SEM - Zeiss NVISION 40 dual beam FIB machine, equipped with a high resolution SEM Gemini column) enabled to investigate films morphologyA suitable procedure for sample preparation for cross-sectional observations was set up because the flexible nature of the Kapton substrate avoids to employ standard cutting procedures to obtain a clean, sharp and smooth sample cross-section. We therefore scratched the AlN film from the substrate by using a bistoury blade and transferred the removed material to a carbon tape. All the procedure is performed under optical microscope. The large number of film pieces randomly scattered on the substrate guarantees the availability of fragments in the proper orientation for cross sectional analysis, whereas their sticking to carbon tape guarantees the mechanical stability, necessary for high resolution investigations.

### Electrical and mechanical characterization of AlN thin film

The dielectric constant of an AlN thin film inserted between two Ti electrodes 150 nm-thick was evaluated through C-V measurements. Top electrodes were circular-shaped with a diameter of 200 μm, realized by photolithography process. This stacked structure was sputtered on Si/SiO_2_ substrate. The employed frequencies to perform C-V measurements ranged from 20 Hz to 1 MHz.

Young modulus of AlN thin film was evaluated by nanoindentation^33^ by employing a Nano Indenter *TTX – NHT2 CSM Instruments* (*Nanoindentation & Micro scratch platform*) equipped with a diamond Berkovich tip. Mechanical properties were evaluated when varying the applied load in the range 0.6 mN – 4 mN (3 measurements per each load value). This enabled to stady the variation of the mechanical properties through the composite film-substrate. During an indentation test, the load is ramped up to a selected peak value (*P*_max_) at a constant loading. The penetration depth represents the maximum displacement reached at the end of the loading phase in correspondence of P_max_. The Oliver and Pharr method^34^ was employed to extract Young modulus value from load and displacement.

### Device fabrication and characterization

**Figure 9:**
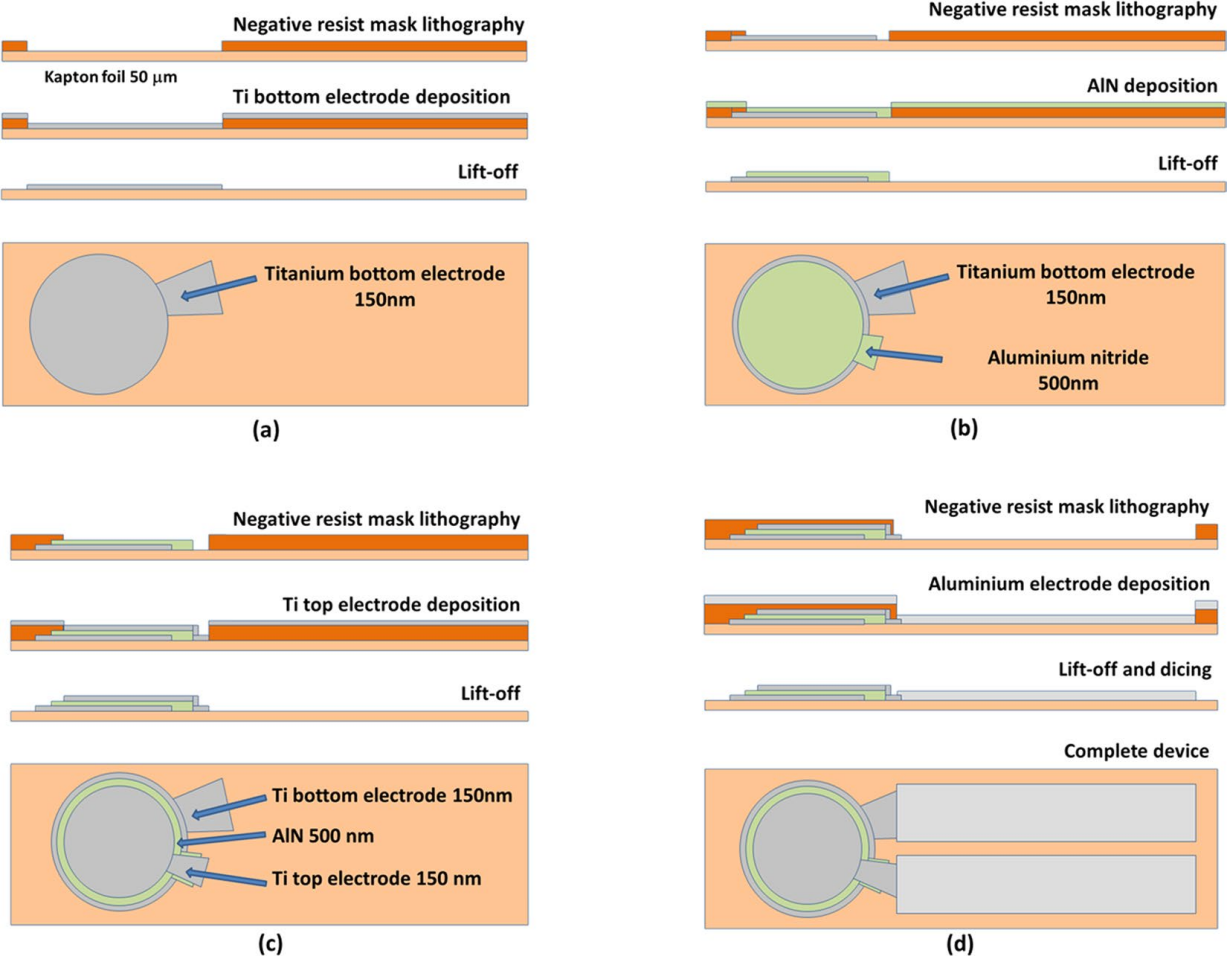
Fabrication process of the AlN-based flexible pressure sensor realized on Kapton substrate: (**a**) bottom electrode fabrication, (**b**) AlN active layer deposition, (**c**) top electrode deposition, (**d**) aluminum contacts (complete device).

In order to overcome typical limitations imposed by flexible substrate in terms of flatness and critical dimension control, conventional cleanroom processes (i.e. photolithography and etching techniques) were optimized for device fabrication. To enhance the reliability of the fabrication steps, the 50 μm thick Kapton foil was glued on 4″ silicon substrate through a PDMS adhesive layer prior the lithography process. The pressure sensor has a circular geometry with the AlN layer (500 nm-thick) interposed between two Ti electrodes (150 nm-thick) in a stacked concentric configuration. The diameter is imposed by the device housing related to needle footprint. Specifically, the system should be mounted between the shaft of a linear actuator and the needle, as previously described. The top and bottom electrode diameters are 3.8 mm and 4 mm, respectively. The fabrication process starts with the Kapton substrate cleaning in acetone and 2-propanol at room temperature, followed by 10 minutes oxygen plasma at 600 mTorr/800 W, and subsequent dehydration onto hotplate. The different fabrication steps are reported in Fig. 9: the bottom Ti contact has been realized with a 150 nm Ti deposition onto a 2 μm thick AZ nLOF2020 resist mask, and subsequent lift-off procedure (Fig. 9a). The second and third layer lithography steps were required for the definition of AlN active layer (500 nm) and the top Ti electrode (150 nm), (Fig. 9b,c. The devices were designed as stacked circular concentric structures to preserve the circular symmetry and optimized the generated charge; a 300 nm thick aluminum pads have been deposited on titanium contacts for final bonding and packaging (Fig. 9d). After all fabrication steps (thin films deposition and lithography), the Kapton substrate was mechanically detached from silicon support and diced to obtain final devices.

**Figure 10:**
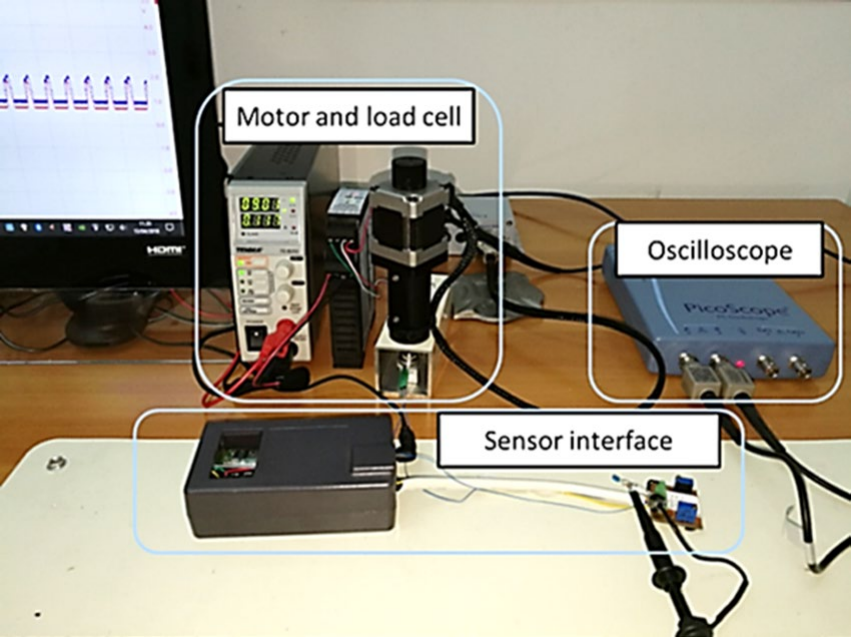
Custom designed experimental setup for the characterization of the pressure sensor.

The characterization of the sensor has been performed by using the experimental custom set-up, we developed and represented in Fig. 10.

**Figure 11:**
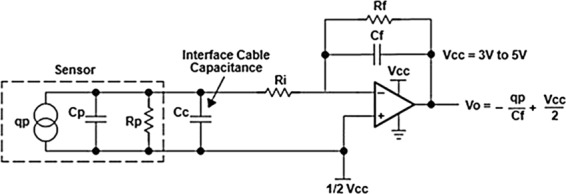
Charge mode amplifier circuit realized for the sensor interface.

It consisted of a PI Instruments M-229.26S high precision stepper linear actuator, a 500 gr calibrated load cell, a custom designed sensor interface and a 2 channel, 100 MHz oscilloscope. The stepper actuator is equipped with a controller and a proprietary software to move a rubber rod at a specific speed and applied force on the sensor. The load cell detected the force applied on the sensor surface and the sensor interface converts the charge coming from the pressure sensor to voltage. The sensor interface was realized by using the charge mode amplifier circuit shown in Fig. 11.

It amplifies and converts to voltage the charges generated when the sample is mechanically deformed according to the following equation: ${q}_{p}={C}_{f}\cdot (\frac{{V}_{cc}}{2}-{V}_{0})$ where *q*_*p*_ is the charge generated by the piezoelectric material, *C*_*f*_ is the capacitance of the capacitor, *V*_0_ is the output of the charge amplifier and *V*_*CC*_ is the power supply voltage of the interface. The realized circuit requires few electronic components and it is based on the low power, small dimension, low noise, LinCMOS Rail-to-Rail Texas TLC227X Operational Amplifier. Therefore, it is possible to obtain high performance in terms of accuracy, power consumption and area occupation. This is a relevant aspect both of the sensor characterization and for the successive integration of the readout circuit in the sensor chip. The oscilloscope collected and recorded the data. This experimental set-up permitted to evaluate the piezoelectric constants of AlN thin film by the quasi-static method^35^. Before performing the measurements, the error of the set-up was evaluated. Therefore, the aforementioned characterization procedure was accomplished to a reference sample of known piezoelectric coefficient; specifically, in this work, a commercial PZT disc (#APC-842 from AmericanPiezoCeramics, International LTD) was used. The piezoelectric coefficient of the reference has been calculated in the same experimental conditions of the fabricated piezoelectric pressure sensor and the evaluated measurement uncertainty was equal to 6%, according to^36^.

### FEA simulations (COMSOL Multiphysics)

A coupled piezoelectric-circuit FEA by using Comsol Multiphysics software enabled to study the performance of the AlN pressure sensor directly connected to a signal conversion and amplification circuit. The direct piezoelectric effect was simulated on a 3D model of the AlN based pressure sensor, in response to a normal load applied onto the top electrode. In stress-charge form, the relation between strain S, stress T, electric field E and electric displacement field D is given by:

$$\begin{array}{rcl}{\boldsymbol{T}} & = & {c}_{E}{\boldsymbol{S}}-{e}^{T}{\boldsymbol{E}}\\ {\boldsymbol{D}} & = & e{\boldsymbol{S}}+{\varepsilon }_{S}{\boldsymbol{E}}\end{array}$$

where *c*_*E*_, *e* and *ε*_*S*_ are the elasticity matrix, the coupling matrix and the relative permittivity matrix of the piezoelectric material (subscripts indicate values at *E* and *S* constant). The materials properties used for simulations are given in Table 1.

**Table 1:** Materials properties used for simulations.

Materials properties	
Kapton substrate^37^	ρ = 1.42 g/cm^3^; E = 3.10 GPa; ν = 0.34
AlN^38^	ρ = 2.70 g/cm^3^; ε_ii_ = 9, ε_ij_ = 0 *Elasticity matrix coefficients (GPa*) C_11_ = C_22_ = 345 C_12_ = 125 C_13_ = C_23_ = 120 C_33_ = 395 C_44_ = C_55_ = 118 C_66_ = 110 *Coupling matrix coefficients* (*C/m*^2^) e_24_ = e_15_ = −0.48 e_31_ = e_32_ = −0.58 e_33_ = 1.55
Ti^39^	ρ = 4.51 g/cm^3^; E = 100 GPa; ν = 0.34

The bottom surface of the Kapton substrate was assumed fully constrained (that is, the displacements are zero in all directions), being stuck to the load cell holder in the experiments. The other surfaces of the structure were set free.

## Conclusions

The fabrication and characterization of an event detection subsystem composed of a flexible piezoelectric pressure sensor and the electronic interface to be integrated into an implantable artificial pancreas (IAP) for diabetic patients have been presented. A such device has been requested to monitor one of the actions involved in the artificial organ operation. In particular, the developed subsystem is located at the connection of a needle with a linear actuator with the aim to reliably detect the occurred punching of an ingestible insulin-filled capsule for the following insulin transfer the an implanted reservoir. The final sensor device, operating in d_33_ mode, is made of on AlN thin film sandwiched between two Ti electrodes, sputtered on Kapton substrate which guarantees the flexibility. The characterization, by using the quasi-static method, proved the reliability of such fabricated sensor, which is able to measure quasi-static force up to 4 N in a range of very low speeds (2 mm/s–3 mm/s) of the actuator where the sensor is located. The system is able to correctly detect the punching events, with a Signal-to-Noise Ratio (SNR) of 6.21. The flexibility, together with the biocompatibility and self-powering of the proposed sensor, are important characteristics in the biomedical field because implanted devices are often required to be directly integrated with soft tissue/organs and to be able to harvest energy from natural sources around the patient. These properties and the enabling technology presented in this paper may enable and encourage in the near future the employment of piezoelectric pressure sensors into implantable artificial organs.
